# A Prenatal Diagnosis of Verheij Syndrome in a Fetus Harboring a de novo *PUF60* Variant

**DOI:** 10.1002/ccr3.71230

**Published:** 2025-10-15

**Authors:** Catia Mio, Maria Orsaria, Alessandra Franzoni, Elisa Bregant, Elena Betto, Jessica Zucco, Eliana Demori, Giuseppe Damante, Flavio Faletra

**Affiliations:** ^1^ Department of Medicine University of Udine Udine Italy; ^2^ Institute of Pathology University Hospital of Udine Udine Italy; ^3^ Institute of Medical Genetics University Hospital of Udine Udine Italy

**Keywords:** exome sequencing, polymalformative syndrome, prenatal diagnosis, PUF60, spliceosomopathy, Verheij syndrome

## Abstract

This case illustrates how trio‐based exome sequencing can uncover de novo Poly‐U‐binding splicing factor 60‐KD (*PUF60*) gene variants responsible for Verheij syndrome (VRJS) in utero, even in the absence of classic features such as growth restriction. Our findings broaden the prenatal phenotype and underscore the role of advanced genomic tools in evaluating polymalformative syndromes.

## Graphical abstract

Here, we report the case of a fetus affected by multiple congenital anomalies (MCA) syndrome detected during the second trimester through ultrasound scan, in which a *de novo* pathogenetic variant in the *PUF60* gene (NM_078480.3:c.382_383del; p.Met128ValfsTer11) was identified by trio‐based exome sequencing. Postmortem examinations after a voluntary termination of pregnancy evidenced a complex polymalformative pattern characterized by the presence of craniofacial and visceral abnormalities. The assessed phenotype seems consistent with a diagnosis of VRJS.

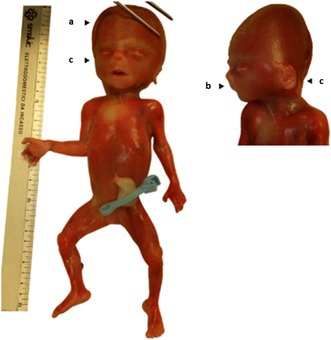

## 1 Introduction

Verheij syndrome (VRJS, MIM #615583) is a rare genetic spliceosomopathy with a wide phenotypic spectrum. It is mostly characterized by growth restriction, delayed psychomotor development, dysmorphic craniofacial features, microcephaly, and skeletal abnormalities [1, 2]. Less frequent features include coloboma, congenital abnormalities of the heart, kidney, and urinary tract [3].

Initially, the deletion of the 8q24 chromosome region, typically spanning from 78 kb to 1 Mb, with a common minimum region encompassing *SCRIB*, *NRBP2*, and *PUF60* genes, [4] was considered as the disease‐causing genetic abnormality. Recently, the identification of pathogenic variants in *PUF60* in individuals with comparable phenotypes has established that the loss of function of this gene is responsible for the VRJS.

The poly(U)‐binding‐splicing factor (*PUF60*) gene encodes an mRNA‐binding protein in several nuclear processes, such as pre‐mRNA splicing, apoptosis, and transcriptional regulation, through interaction with other proteins [3, 5, 6].

PUF60 directly interacts with splicing factor 3B, subunit 4 (SF3B4), and facilitates the recognition of the 3′ splice site by interacting with U‐rich tracts. Moreover, it plays a crucial role in the recruitment of U2 and U5 small nucleolar ribonucleoprotein to the intronic sequences for splicing [7, 8]. In addition to splicing facilitation, PUF60 is able to control the transcription of several genes [9, 10]. *PUF60* haploinsufficiency leads to a spectrum of disorders associated with spliceosome functional deficiency. Heterozygous loss‐of‐function (LoF) variants of the *PUF60* gene are associated with VRJS phenotypes due to defective spliceosomal function and aberrant mRNA maturation. Notwithstanding the majority of PUF60 variants cause haploinsufficiency, no clear genotype–phenotype correlations have yet been reported.

Differential diagnoses for VRJS include other genetic syndromes, particularly those involving congenital anomalies and developmental delays. Notably, Cornelia de Lange syndrome, Noonan syndrome, Coffin‐Siris syndrome (CSS), and Rubinstein‐Taybi syndrome (RSTS) affected patients share several phenotypic abnormalities with VRJS [11]. It is worth noting that, despite *PUF60* variants not being associated with a unique DNA methylation pattern in patients' blood, a pattern resembling the episignature of Cornelia de Lange syndrome (CdLS) has been identified, suggesting a convergent pathway for *PUF60* and CdLS‐related genes that has not yet been yet elucidated [4]. Consistent with this finding, some patients with a clinical suspicion of CdLS turned out to harbor a pathogenic *PUF60* variant, further emphasizing the phenotypical overlap between these two syndromes [3, 12].

So far, about 80 patients with VRJS have been published with slightly different multisystem features [4, 12, 13, 14, 15]. Both nonsense, frameshift, splice‐site, and missense *PUF60* variants have been identified to be responsible for VRJS. For the majority of them, postnatal diagnosis has been made combining clinical phenotyping with next‐generation sequencing analysis. Nevertheless, Wells et al. and Miao et al. reported *PUF60* alterations by post‐mortem molecular evaluations in fetuses who underwent either spontaneous or voluntary termination of pregnancy. Indeed, a heterozygous *PUF60* LoF variant was identified in a fetus spontaneously aborted in utero at 28 gestational weeks (GW) presenting with congenital heart defects (absence of pulmonary valves, left and right pulmonary artery dilatation, ventricular septal defect, aortic straddle, cardiac enlargement) and preaxial polydactyly of the left thumb [6]. Furthermore, a 985 kb deletion in chromosome 8q24.3, encompassing 42 genes including *PUF60*, has been identified in a fetus showing intrauterine growth restriction (IUGR) and cardiac malformation, after elective termination of pregnancy [16]. Here, we report the case of a fetus affected by multiple congenital anomalies (MCA) syndrome, in which a de novo *PUF60 LoF* variant was detected during pregnancy by a trio‐based WES. This is the first case of a prenatal diagnosis of VRJS due to a *PUF60* pathogenic variant.

## 2 Case History/Examination

The proband was a male fetus from healthy nonconsanguineous parents. The family history was negative for stillbirths, intellectual disability, or birth defects. No malformations were detected on the first trimester ultrasound scan at 12 + 3 gestational weeks (GW). The nuchal translucency was measured at 1.64 mm. Non‐invasive prenatal testing (NIPT) showed no evidence of fetal aneuploidy (fetal fraction: 8%). During the second‐trimester ultrasound (20 + 3 GW), a right aortic arch with the ductus arteriosus positioned left of the trachea and a possible subaortic defect of the interventricular septum were observed. Invasive prenatal testing was then recommended, and amniocentesis was performed at 21 + 0 GW.

## 3 Methods

### 3.1 Editorial Policies and Ethical Considerations

Informed consent to molecular analysis, publication of photographs and clinical information was obtained according to the Helsinki Declaration. Invasive prenatal testing, genetic testing, and post‐mortem evaluations were conducted at the Azienda Ospedaliera Universitaria Friuli Centrale in Udine (Italy). Written informed consent for the publication of this case report was obtained from the patient's parents.

### 3.2 Conventional Chromosome Analysis

Chromosome analysis was performed according to standard procedures using QFQ banding technique at 400 band resolution. Metaphase spreads were prepared from phytohaemagglutinin‐stimulated lymphocytes, cultured at 37°C for 72 h.

### 3.3 DNA Extraction

Genomic DNA was extracted from cultured amniocytes or parents' peripheral blood samples collected into 10 mL EDTA K2 blood collection tubes using the DNeasy Blood & Tissue DNA Kit (Qiagen, Hilden, Germany), according to the manufacturer's instructions. DNA quantity was estimated using the Qubit dsDNA HS Assay Kit on a Qubit 4.0 Fluorometer (Thermo Fisher Scientific, Waltham, MA, USA).

### 3.4 Chromosome Microarray Analysis (CMA)

Array CGH analyses were performed using the Agilent Human Genome CGH oligonucleotide array 180 K (Agilent Technologies, Santa Clara, CA, USA) as previously described [17]. Images were analyzed with the Agilent Feature Extraction, Genomic Workbench 6.5.018 Lite Edition Software, and genomic coordinates were evaluated according to GRCh37/hg19.

### 3.5 Exome Sequencing (ES)

Barcoded libraries were generated from 50 ng of DNA and the exonic regions and flanking splice junctions (±25 bp flanking each exon) of about 22,000 coding genes were captured using the WholEX pro sequencing kit (4bases SA, Manno, Switzerland). Sequencing was performed in paired‐end 2 × 150 bp on a NextSeq system (Illumina Inc., San Diego, CA, USA). Reads were aligned to human genome build GRCh38/hg38, and variant calling, annotation, and classification were performed with eVai (enGenome, Pavia, Italy). A minimum depth coverage of 20× and a minimum alternate allele frequency of 20% (VAF ≥ 20%) were considered suitable for analysis. Variants with frequency < 0.1% in population‐based databases (i.e., gnomAD), exonic missense, splicing, stop‐gain, stop‐loss, and frameshift insertion and deletion variants were retained for further evaluation. The following public databases were used for variant interpretation: HGMD Professional (https://my.qiagendigitalinsights.com/bbp), ClinVar (https://www.ncbi.nlm.nih.gov/clinvar/), Varsome Premium (https://varsome.com/). Variants were classified according to the American College of Medical Genetics and Genomics (ACMG) guidelines [18, 19]. Candidate variants were classified as potentially disease‐causing based on ACMG criteria, reports of previous cases with a comparable phenotype, animal models, and/or functional studies. Sanger sequencing was performed to confirm relevant results obtained by ES.

## 4 Conclusion and Results

**FIGURE 1:**
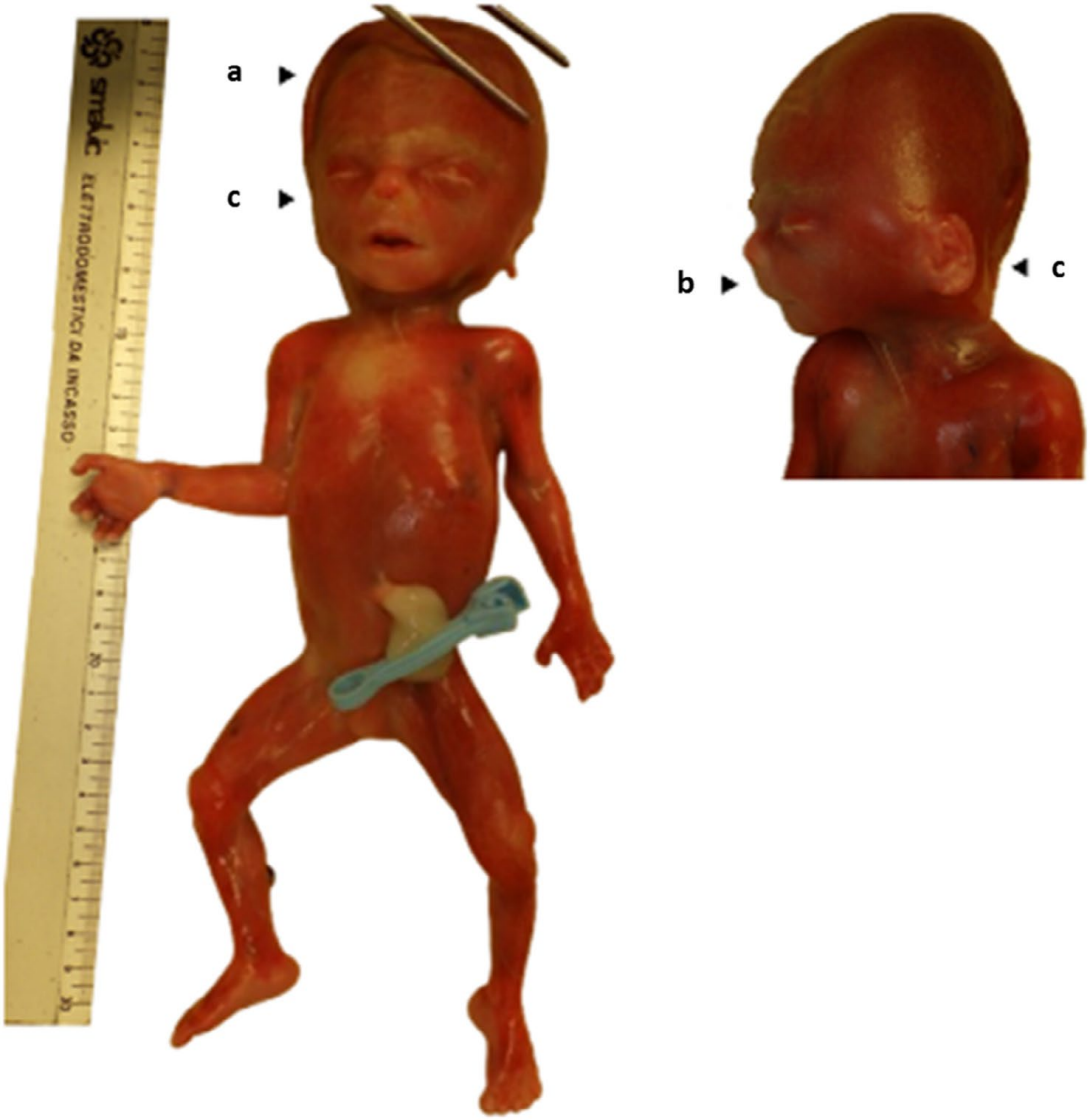
Major craniofacial abnormalities detected in our proband. Body view (left) and face (right). Despite the early gestational age, the fetus presented some of the typical features of VRJS such as skull anomalies (turricephaly, arrowed—a), hypertelorism, short nose with anteverted nares, long nasolabial filter (arrowed—b), and low‐set malformed ears (arrowed—c).

Due to the pattern of malformations, conventional chromosome analysis and chromosomal microarray analysis (CMA) together with trio‐based exome sequencing (ES) were performed. Aortic arch duplication, corpus callosum dysplasia, hypoplastic kidneys, small filling stomach, and failure to visualize the gallbladder were assessed during a level II ultrasound examination at 22 + 2 GW. No structural variants or micro‐deletions/duplications were detected by chromosome analysis and CMA (46, XY). ES analysis was performed, and after filtering low‐quality data and benign and likely benign variants, 9 *de novo* variants in 8 genes were identified. Notably, a de novo pathogenic variant in *PUF60* (NM_078480.3):c.382_383del; p.(Met128ValfsTer11) was highlighted. Sanger sequencing (Figure S1) confirmed the presence of the *PUF60* pathogenic variant in fetal DNA and its absence in parents' DNA. Parents decided on an elective termination of pregnancy at 22 + 4 GW, and the fetus was delivered by induced labor. Autopsy revealed a male fetus consistent with 22 weeks of gestation, with multiple craniofacial and visceral abnormalities (Figure 1).

**FIGURE 2:**
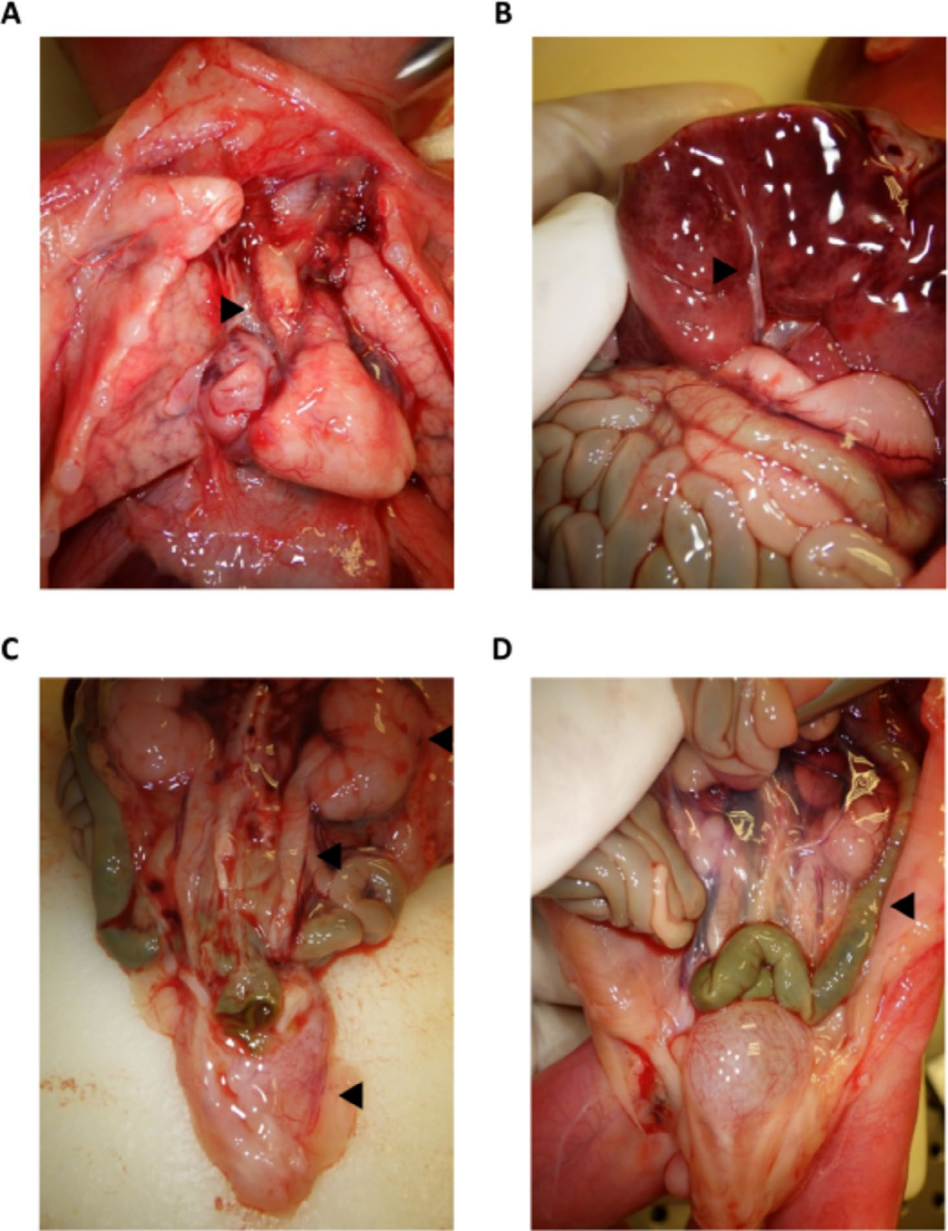
Major visceral abnormalities detected in our proband. (A) Straightened right aortic arch (arrowed). (B) Gallbladder and extrahepatic biliary tract hypoplasia (arrowed). (C) ureters and urinary bladder ectasia, bilateral kidney hypoplasia (arrowed). (D) abnormality of intestinal rotation and mesenteric attachment of the sigmoid colon (arrowed).

Furthermore, histological examinations showed hypercontraction bands in skeletal muscles, consistent with a terminal acute distress, as well as blood congestion and acute polyvisceral hemorrhage, likely related to the induced abortion. Craniofacial dysmorphisms included turricephaly, hypertelorism, short nose with anteverted nares, long nasolabial filter, and low set malformed ears. Visceral malformations comprised a straightened right aortic arch (Figure 2A) in a suggestive picture for mirror branching of the supra‐aortic vessels with associated ventricular septal defect, esophageal atresia with double fistula, gallbladder (Figure 2B), and extrahepatic biliary tract hypoplasia, bilateral kidney hypoplasia (length of 17 mm) (Figure 2C), intestinal malrotation (Figure 2D), and umbilical vein dilatation. Moreover, phase contrast microscopic evaluation of placental tissue showed villous dysmorphic features suggestive of a genetic abnormality and histological signs of shallow placental implantation.

## 5 Discussion

To date, diagnosis of Verheij syndrome relies on a combination of clinical assessments and genetic testing since VRJS shows phenotypic similarities with other genetic syndromes such as Cornelia De Lange, Coffin‐Siris, and Rubinstein‐Taybi syndromes [8]. Given the paucity of prenatal findings of VRJS, exome sequencing plays a pivotal role in establishing an accurate diagnosis.

In this study, using trio‐ES, we identified a de novo variant in *PUF60* (NM_078480.3):c.382_383del; p.(Met128ValfsTer11) in a fetus presenting multiple malformations during the level II ultrasound scan at 22 + 2 GW. The frameshift variant is located in exon 6 of 12 and is predicted to result in a premature stop codon. This variant disrupts a critical protein region, such as the first RNA recognition motif domain (RRM1), involved in RNA binding.

Postmortem findings were consistent with the diagnosis of VRJS, i.e., facial dysmorphisms, cardiac and renal abnormalities. Albeit, the developmental pattern resulted consistent with a fetus of 22 + 2 GW, which differs from previous reports where IUGR is a prominent feature [4, 14, 16]. Notably, in our case microcephaly was absent, despite being a frequent characteristic in VRJS patients [13].

Notwithstanding, our patient presented several clinical features suggestive of VRJS and overlapping with previously reported cases [13, 20]. Table 1 summarizes the phenotypic characteristics of our patient compared with previous reports [3, 4, 6, 8, 13, 20].

**TABLE 1:** Phenotypic comparison between our patients and the previously reported VRJS‐affected patients.

Clinical characteristics	Previous cases (%)	Our case
Growth delay		
Intrauterine growth restriction	30	−
Short stature	90	+
Feeding difficulties	60	n.a.
Craniofacial features		
Dysmorphyc features	70	+
Oral/palatal abnormalities	60	n.a.
Craniofacial anomalies	60	+
EAR features		
Hearing loss	50	n.a.
Low‐set ears	40	+
Other ear structural anomalies	10	+
Eye features		
Coloboma	60	n.a
Other ophthalmological abnormalities	30	n.a
Skeletal features		
Microcephaly	35	+
Joint laxity/dislocation	50	−
Vertebral fusion/scoliosis	60	−
Limb anomalies	60	−
Neurological manifestations		
Neurodevelopmental delay	90	n.a
Brain malformations	40	+
Seizures	25	n.a.
Intellectual disability	90	n.a.
Speech delay	60	n.a.
Defects in other organs		
Congenital heart defects	60	+
Gastrointestinal abnormalities	60	+
Congenital anomalies of kidney and urinary tract	25	+
Genital abnormalities	20	−

*Note:* Percentages were calculated based on previously published cases (see [3, 4, 6, 8, 13, 20]).

Abbreviations: −, absent; +, present; n.a., not applicable (i.e., not evaluated).

Although some clinical features, such as phocomelia, are specific for CdL, others are shared between these two syndromes, including, cardiac and genitourinary malformations, auditory abnormalities, and oral/palatal deformities. More interestingly, also some facial dysmorphisms present in fetuses with CdLS [21] are similar to those detected in our proband. Indeed, the synophrys, the short nose with anteverted nostrils and depressed nasal bridge, the long philtrum with thin upper lip, the micrognatia, and the low‐set ears are present in this fetus and in CdLS patients. These findings support a potential connection between *PUF60* and CdLS‐related genes with convergent pathways, as suggested by Hoogenboom et al. [4]. They, demonstrated that a VRJS patient exhibited a methylation profile resembling the one of CdLS, consistent with the phenotypic similarities [12]. A critical question is how *PUF60* haploisufficiency contributes to developmental anomalies in VRJS. PUF60 interacts with SF3B4, thereby facilitating recognition of the 3′ splice site. A subset of the developmental anomalies identified in fetus described in this research may result from reduced PUF60‐SF3B4 interaction. Indeed, patients carrying *SF3B4* mutations share clinical features with VRJS, particularly, cardiac and craniofacial malformations [22, 23].

In conclusion, we identified a *PUF60* variant (NM_078480.3):c.382_383del; p.(Met128ValfsTer11) in a fetus presenting multiple congenital anomalies. The identification of a pathogenic *PUF60* variant, despite negative chromosome microarray and karyotyping, highlights the indispensable role of exome sequencing analysis in modern prenatal diagnostics. This approach is particularly valuable in contexts of diagnostic uncertainty or overlapping syndromic features such as VRJS.

## Author Contributions

**Catia Mio:** data curation, formal analysis, methodology, writing – original draft, writing – review and editing. **Maria Orsaria:** data curation, formal analysis, methodology. **Alessandra Franzoni:** data curation, formal analysis, methodology. **Elisa Bregant:** data curation, formal analysis, methodology. **Elena Betto:** data curation, formal analysis, methodology. **Jessica Zucco:** data curation, formal analysis, methodology. **Eliana Demori:** data curation, formal analysis, methodology. **Giuseppe Damante:** conceptualization, writing – review and editing. **Flavio Faletra:** data curation, formal analysis, methodology, writing – original draft, writing – review and editing.

## Ethics Statement

Exome sequencing, conventional chromosome analysis, and chromosome microarray analysis were performed as clinical services. Written informed consent was obtained from both parents for molecular analysis, publication of clinical data, and postmortem images in accordance with the ethical guidelines of the Helsinki Declaration.

## Conflicts of Interest

The authors declare no conflicts of interest.

## Supporting information


**Figure S1:** Sanger sequencing results. Electropherograms showing the de novo occurrence of the PUF60 variant (harrowed).

## Data Availability

The data that support the findings of this study are available from the corresponding author upon reasonable request.
