# Supplementary figures and images for: A Prenatal Diagnosis of Verheij Syndrome in a Fetus Harboring a de novo PUF60 Variant
Source: Clin Case Rep. 2025 Oct 15;13(10):e71230. doi: 10.1002/ccr3.71230 (PMC12527814; doi:10.1002/ccr3.71230)

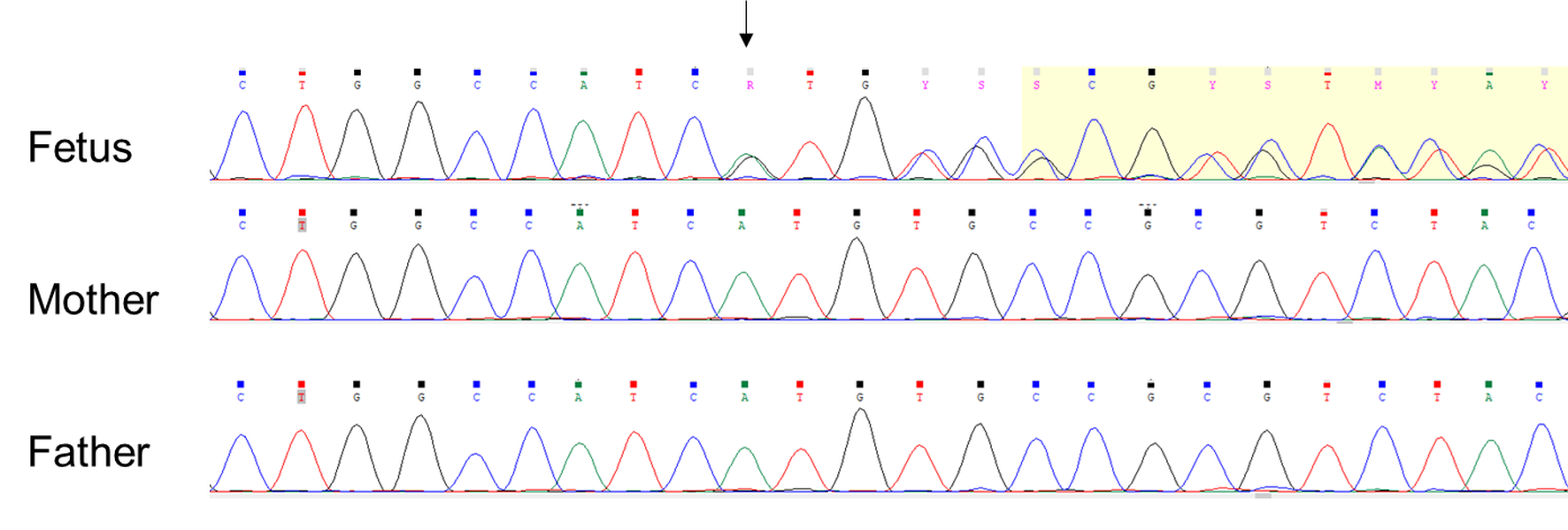

Supplement: Supplementary file 1 — Figure S1: Sanger sequencing results. Electropherograms showing the de novo occurrence of the PUF60 variant (harrowed). [file CCR3-13-e71230-s001.tif]
